# Newborn Screening Practices for Beta-Thalassemia in the United States

**DOI:** 10.3390/ijns7040083

**Published:** 2021-12-13

**Authors:** Michael A. Bender, Mary Hulihan, Mary Christine Dorley, Maria del Pilar Aguinaga, Jelili Ojodu, Careema Yusuf

**Affiliations:** 1 Clinical Research Division, Department of Pediatrics, Fred Hutchinson Cancer Research Center, University of Washington, Seattle, WA 98109, USA; mbender@fredhutch.org; 2 National Center on Birth Defects and Developmental Disabilities, Centers for Disease Control and Prevention, Division of Blood Disorders, Atlanta, GA 30329, USA; mhulihan@cdc.gov; 3 Tennessee Department of Health Laboratory Services, Nashville, TN 37243, USA; M.Christine.Dorley@tn.gov; 4 College of Health Professions, School of Health Sciences, Walden University, Minneapolis, MN 55401, USA; 5 Department of Obstetrics and Gynecology, Meharry Medical College, Nashville, TN 37208, USA; maguinaga@mmc.edu; 6 Meharry Sickle Cell Center, Meharry Medical College, Nashville, TN 37208, USA; 7 Association of Public Health Laboratories, Silver Spring, MD 20910, USA; careema.yusuf@aphl.org

**Keywords:** newborn screening, beta thalassemia, hemoglobinopathies, isoelectric focusing, high performance liquid chromatography, harmonization, standardization

## Abstract

Beta-thalassemia, a heritable condition of abnormal hemoglobin production, is not a core condition on the United States Recommended Uniform Screening Panel (RUSP) for state and territorial newborn screening (NBS) programs. However, screening for sickle cell disease (which is on the core RUSP) also detects reduced or absent levels of hemoglobin (Hb) A and certain other Hb variants associated with beta-thalassemia and, thus, allows for a timely referral to appropriate healthcare to minimize sequalae of the disease. The Association of Public Health Laboratories’ Hemoglobinopathy Workgroup administered a comprehensive survey of all U.S. NBS programs to assess beta-thalassemia testing methodologies, the cutoffs for defining beta-thalassemia major, and the reporting and follow-up practices. Forty-six (87%) of the programs responded. Thirty-nine of the 46 responding programs (85%) report some form of suspected beta-thalassemia; however, the screening methods, the percentage of Hb A used as a cutoff for an indication of beta-thalassemia major, and the screening follow-up vary widely. The standardization of technical and reporting procedures may improve access to specialty care prior to severe complications, increase genetic counseling, and provide data needed to better understand the public health impact and clinical outcomes of beta-thalassemia in the United States.

## 1. Introduction

Thalassemias are hereditary hemolytic anemias that stem from mutations altering the normal 1:1 ratio of alpha- to beta-globin chains, necessary for normal hemoglobin (Hb) A assembly, resulting in microcytosis and anemia. Almost 300 beta-thalassemia mutations of varying severity that can occur on one or both alleles have been described. This results in a continuum of phenotypes from clinically insignificant to the total absence of gene expression and severe disease. The anemia and resulting ineffective erythropoiesis of beta-thalassemia can vary from mild laboratory abnormalities with no impact on morbidity to transfusion dependence as well as severe and life-threatening complications, including growth delays, boney abnormalities and deformities, splenomegaly, iron overload and the resultant liver, heart and endocrine tissue damage, and an increased thromboembolic risk, among other complications.

Individuals with beta-thalassemia major, a severe transfusion-dependent hemolytic anemia, may experience irreversible bone and organ damage and the sequalae of iron overload if untreated [1]. Individuals with beta-thalassemia intermedia do not require regular transfusions to survive but still have significant complications and, if not treated, can have worse outcomes than transfused patients with beta-thalassemia major. Individuals with beta-thalassemia minor (thalassemia trait) may have a mild hypo-chromic, microcytic anemia, often resulting in a misdiagnosis of iron deficiency and inappropriate treatment with iron, while without there being significant clinical sequelae, there are major genetic counseling implications. Compound heterozygote states of a beta-thalassemia allele with a hemoglobin variant that also results in reduced beta-globin synthesis, such as Hb E/beta^0^-thalassemia, can lead to a transfusion-dependent thalassemia-major phenotype.

Approximately 0.6% of the global population are carriers of at least one beta-thalassemia mutation [2]. The prevalence of beta-thalassemia is highest among Southeastern and Southern Asian populations, as well as those from the Middle East, Mediterranean countries, and North and Central Africa. There is little information about the epidemiology of beta-thalassemia in the United States outside of California, where the birth prevalence of beta-thalassemia major and compound heterozygotic states (excluding sickle/beta-thalassemia) was calculated to be 2.3 per 100,000 births between 2001 and 2011 [3].

While testing for beta-thalassemia is not part of the United States (U.S.) core Recommended Uniform Screening Panel (RUSP) (https://www.hrsa.gov/advisory-committees/heritable-disorders/rusp/index.html, (accessed on 30 November 2021)) for the 53 state and territorial newborn screening (NBS) programs, the methodologies used for the detection of sickle cell disease, which is a core RUSP condition that is performed by all programs, are also capable of detecting a significantly diminished ratio of Hb A to fetal Hb (Hb F). This diminished ratio is suggestive of beta-thalassemia, as well as certain other hemoglobin variants that can lead to a beta-thalassemic phenotype, which allows for the detection of babies with clinically severe beta-thalassemia. Epidemiologic studies are complicated as distinguishing beta-thalassemia major from beta-thalassemia intermedia is done on a phenotypic basis. Thus, NBS laboratory results do not always allow for the determination of the form of beta-thalassemia. Early detection and reporting may allow for early access to specialty care and the initiation of transfusions before the development of clinical sequalae, thereby avoiding the physical stigmata and morbidity stemming from chronic anemia [4]. To address the lack of knowledge about beta-thalassemia screening practices among NBS programs in the United States, the Association of Public Health Laboratories’ (APHL) Hemoglobinopathy Workgroup administered a comprehensive survey of NBS programs’ beta-thalassemia testing methodologies, their cutoffs to define beta-thalassemia major, and their reporting and follow-up practices.

## 2. Materials and Methods

APHLs’ Hemoglobinopathy Workgroup initiated a nationwide survey of U.S. NBS programs in August 2018. A ten-question survey was emailed to 53 U.S. NBS programs (50 states, District of Columbia, Guam, and Puerto Rico) (Survey Questions in the Supplementary Materials). Non-responding state NBS programs received reminder emails and telephone calls in an effort to maximize the response rate. The email, which was addressed to the main contacts at each NBS program (i.e., laboratory directors, laboratory managers, and follow-up coordinators), was sent with a survey link, encouraging collaboration to complete one survey per NBS program. Questions covered the methods used for testing, procedures for the reporting of results, and follow-up protocols.

## 3. Results

A total of 46 (87%) of 53 programs responded to the survey (Table S1). All reporting programs perform some form of screening for beta-thalassemia. Nine (20%) of the responding programs use only one screening method: high performance liquid chromatography (HPLC) *n* = 4; isoelectric focusing (IEF) *n* = 5. Of the 37 programs using two screening methods, 25 (68%) use IEF followed by HPLC; eight (22%) use HPLC followed by IEF; and four (11%) use IEF followed by DNA mutation analysis for hemoglobin variants. Thirty-nine (85%) of the responding programs report at least some form of potential beta-thalassemia, with 29 (74%) of these referring newborns with possible beta-thalassemia major for a definitive diagnosis. Most of these programs (25/29; 86%) reported using specific thresholds for detecting possible beta-thalassemia major. Sixteen programs define beta-thalassemia major as “Hb F-only” or <1% Hb A. Six programs define beta-thalassemia major using other Hb A thresholds, ranging from ≤1% to <3% Hb A. The responses from the three remaining programs could not be interpreted. All but one of the NBS programs that report beta-thalassemia results also provide recommendations for retesting/follow-up.

Twenty-one (54%) of the programs that report at least some form of potential beta-thalassemia do so for potential Hb E/beta^0^-thalassemia results. Twenty-two of the programs (56%) report Hb S/beta^0^-thalassemia, and 30 programs (77%) report Hb E/beta^+^-thalassemia. Two (10%) programs share these results with their state’s NBS follow-up team only; seven (33%) programs share these results with a physician only; 12 (57%) programs share these results with both their NBS follow-up team and a physician. One of the programs that provides Hb E/beta^0^-thalassemia screening results with an NBS follow-up and a physician also sends results to the infant’s parents.

## 4. Discussion

This nationwide survey of NBS programs’ screening and reporting practices for beta-thalassemia shows that reporting 85% of responding state and territorial programs, report at least one form of beta-thalassemia despite the not being on the core RUSP. The survey also shows that there is variation in the screening methods and reporting of the results, which may contribute to state-to-state differences in definitive diagnostics and access to appropriate specialty care. This highlights an opportunity for increased harmonization, which may result in public health benefits and increased health equity.

An infant with beta-thalassemia major will eventually die of heart failure secondary to profound anemia if not transfused. Additional severe complications in children under five include growth and neuro-cognitive deficits and failure to thrive (FTT). Infants with thalassemia intermedia have a more variable clinical phenotype but are at risk for these same complications. If not diagnosed at birth, a definitive diagnosis and intervention can be delayed due to the slowly progressive onset, nonspecific presentation of complications, and not including thalassemia in the differential diagnosis for an infant with growth delay or FTT. This outcome may be accentuated in regions of the United States where thalassemia occurrence and provider awareness are rare. As such, infants may suffer severe clinical complications prior to diagnosis by conventional means. Early diagnosis allows for education, additional health screenings and access to care, such as the appropriate use and timing of transfusions, addressing growth and neurocognitive delays, and the prevention or detection of the onset of complications. In addition, without early detection through NBS, it may take months to obtain an accurate diagnosis, and this may result in a subsequent pregnancy before there is an opportunity for genetic counseling and education.

The guidelines from the Clinical and Laboratory Standards Institute (CLSI) on Newborn Screening for Hemoglobinopathies [5] were developed to help laboratories fulfill their responsibilities with efficiency, effectiveness, and global applicability via the implementation of standards. While the CLSI guideline does not make specific recommendations for reporting out potential beta-thalassemia major, the CLSI algorithm does recommend calling out newborns with screening results with “probable F-only” as having a potential beta^0^-thalassemia, which, with rare exceptions, results in a thalassemia-major phenotype. Thus, this recommendation benefits screen-positive individuals with an early evaluation and diagnosed individuals with likely life-long transfusions and aggressive monitoring.

While infants with no Hb A at birth have beta-thalassemia major, complex genetic influences make it difficult to correlate a specific low level of Hb A at birth to a clinical phenotype. Thus, some infants who display diminished levels of Hb A on the newborn screen may develop beta-thalassemia major but not be reported. Consistent with this, large-scale validation studies have defined cutoffs to define beta-thalassemia major in their systems [6]. In the U.S., multiple modalities (IEF and HPLC) and platforms are utilized for both variant hemoglobin detection and quantification, making validation more complex. Our survey results show that NBS programs use variable thresholds to report out beta-thalassemia, and thus the same newborn at risk for severe complications and referred for evaluation in one state or territory would not be referred in others. While the numbers vary slightly, the theme of there being a low degree of uniformity between NBS programs is similar to what was previously noted for alpha-thalassemia [7]. This variability extends from detection and quantitation to what forms of beta-thalassemia are reported out, to NBS follow-up procedures such as who is notified and how. This presents opportunities for harmonization in testing, reporting, and follow-up that have the potential to improve public health by improving epidemiologic data and providing more equitable access to healthcare.

The importance and impact of screening and detecting beta-thalassemia alleles extends beyond homozygous forms. Although not included in this report, there are beta-thalassemia alleles that, when combined with other mutations of the beta-globin locus, can lead to a variety of clinically significant compound heterozygotic syndromes. The most notable are Hb S/beta-thalassemia, which are not discussed here as they are detected in routine NBS for sickle cell disorders and Hb E disorders. When Hb E but no Hb A is detected on NBS, it could represent Hb E/E, a relatively mild chronic hemolytic anemia, or Hb E/beta-thalassemia. The latter can result in a wide range of phenotypes, including thalassemia major or intermedia. Thus, distinguishing between these diagnoses has large clinical consequences. Despite both potentially leading to similar, severe phenotypes, only 21/39 (54%) of NBS programs reporting beta-thalassemia report Hb E/beta-thalassemia, thus resulting in a potential public health inequity.

A limitation of this work is that seven (13%) NBS programs did not respond to the survey. Although it was determined that two of the nonrespondents contract for specimen testing with NBS programs that did respond to the survey, the numbers presented here may not be generalizable to all NBS programs in the United States.

## 5. Conclusions

An increased uniformity in the screening and referral for the definitive diagnostics and treatment of beta-thalassemia may play a large role in improving timely and appropriate healthcare. An increase in the number of NBS programs reporting beta-thalassemia results for multiple suspected forms of the condition may improve access to specialty care prior to severe complications, increase genetic counseling, and provide data needed to better understand the public health impact and clinical outcomes of beta-thalassemia in the United States. In addition, an increased harmonization of screening and reporting practices will increase equity in at-risk infants and opportunities for families to access medical and genetic counseling services regardless of where a baby is born.

## Supplementary Materials

The following are available online at https://www.mdpi.com/article/10.3390/ijns7040083/s1.
