# Supplementary material for: Newborn Screening Practices for Beta-Thalassemia in the United States
Source: Int J Neonatal Screen. 2021 Dec 13;7(4):83. doi: 10.3390/ijns7040083 (PMC8703506; doi:10.3390/ijns7040083)
Supplement: Supplementary file 1 [file IJNS-07-00083-s001.zip › IJNS-1459548-supplementary.pdf]

**Beta Thalassemia Reporting Survey**

Beta thalassemia is an inherited hemoglobinopathy resulting in a reduction or absence of beta globin chains. Since the expression of beta globin is naturally low during the newborn period, this presents challenges to newborn screening programs on how to interpret reduced or absent Hb A phenotypes.

The purpose of the survey is to gather information and develop a training webinar along with recommendations on screening and reporting of beta thalassemias. Individual program information will be kept confidential. Your participation is vital to assessing the areas where training would be beneficial.

The survey may require input from additional members of your Newborn Screening (NBS) program. Please collate all responses and answer the survey as one NBS program.

Thank you for considering this opportunity to make a meaningful contribution to the hemoglobinopathy community.

**Questions:**

1. Please tell us who you are:
   - First Name, Last Name:
   - Title:
   - Organization:
   - Phone number, email address:

2. What is the hemoglobin screening method and algorithm used by your NBS program? (please select from the options below)
   - Isoelectric Focusing (IEF) followed by High Performance Liquid Chromatography (HPLC)
   - High Performance Liquid Chromatography (HPLC) followed by Isoelectric Focusing (IEF)
   - Isoelectric Focusing (IEF) only
   - Isoelectric Focusing (IEF) using IsoScan for quantitation
   - High Performance Liquid Chromatography (HPLC) only
   - Other, please describe

3. Does your NBS program report any type of beta thalassemia?
   - Yes [go to 3a]
   - No – please explain why {text box} and go to question 9]

   3a. Do you report “possible beta thalassemia major”?
   - Yes [go to 3a1]
   - No [go to 3b]

   3a1. Do you have a cutoff for %Hb A?
   - Yes [go to 3a2]
   - No [go to 3a3]

   3a2. What is your cutoff? (Example: <1%) {text box}

3a3. How is that assessment made? {text box}

3b. Do you report:

| | Yes | No |
|---|---|---|
| Hb C/beta zero thalassemia | | |
| Hb D/beta zero thalassemia | | |
| Hb E/beta zero thalassemia | | |
| Hb S/beta zero thalassemia | | |

**[Go to 3b1 if "Yes" is selected for any row at 3b and go to 3b2 if "No" is selected for any row at 3b]**

3b1. How do you differentiate between homozygous disease:

- Hb C/C and Hb C/beta zero thalassemia
- Hb D/D and Hb D/beta zero thalassemia
- Hb E/E and Hb E/beta zero thalassemia
- Hb S/S and Hb S/beta zero thalassemia

3b2. When you report Hb C/C, Hb D/D, Hb E/E or Hb S/S disease do you state the possibility that a beta zero thalassemia can also be a differential diagnosis? Please explain {text box}

3c. Do you report:

| | Yes | No |
|---|---|---|
| Hb C/beta plus thalassemia | | |
| Hb D/beta plus thalassemia | | |
| Hb E/beta plus thalassemia | | |
| Hb S/beta plus thalassemia | | |

**[Go to 3c1 if "Yes" is selected for any row at 3c and go to 3c2 if "No" is selected for any row at 3c]**

3c1. What is your criteria for differentiating a Hb trait from a beta plus thalassemia (i.e. Hb C vs Hb C/beta plus thalassemia)?

- Hb C and Hb C/beta plus thalassemia
- Hb D and Hb D/beta plus thalassemia
- Hb E and Hb E/beta plus thalassemia
- Hb S and Hb S/beta plus thalassemia

3c2. Please indicate the way Hb X/ beta plus thalassemia is reported (please check all that apply).

- FEA
- FSA
- FCA
- FDA
- Other? Please explain

3d. To whom does your NBS laboratory report these findings? (Please select all that apply)

| | **Physician** | **Parent** | **NBS Follow-Up** | **Other** |
|---|---|---|---|---|
| Hb C/beta zero thalassemia | | | | |
| Hb D/beta zero thalassemia | | | | |
| Hb E/beta zero thalassemia | | | | |
| Hb S/beta zero thalassemia | | | | |
| Hb C/beta plus thalassemia | | | | |
| Hb D/beta plus thalassemia | | | | |
| Hb E/beta plus thalassemia | | | | |
| Hb S/beta plus thalassemia | | | | |

4. Do you use molecular testing for beta thalassemia?
    - Yes [go to 4a]
    - No

4a. What method do you use? (text box)

5. Does your NBS program provide recommendations for patient retesting/follow-up?
    - Yes
    - No

6. Please share any materials and website links you have around beta thalassemia.

7. What information would be helpful for to you to consider or enhance reporting beta thalassemia? (e.g., education, materials?) Please describe {text box}

**The next set of questions ask about NBS activities for hemoglobinopathies in general.**

8. Which common hemoglobin traits do you report? Please select all that apply.
    - Hb S trait
    - Hb E trait
    - Hb D trait
    - Hb C trait
    - Hb O-Arab trait
    - Hb G-Philadelphia trait

8a. To whom are they reported? Please select all that apply

| | **Physician** | **Parent** | **NBS Follow-Up** | **Other** |
|---|---|---|---|---|
| Hb S trait | | | | |
| Hb E trait | | | | |
| Hb D trait | | | | |
| Hb C trait | | | | |
| Hb O-Arab trait | | | | |
| Hb G-Philadelphia trait | | | | |

9. For screening of hemoglobinopathies, how many days post-red blood cell transfusion do you ask for a subsequent screen?
    - 30 days
    - 60 days
    - 90 days
    - 120 days
    - Other, please specify {text box}

10. When screening for hemoglobinopathies, if baby is premature or has low birth weight, do you ask for a second sample?
- Yes [go to 10a]
- No
- Other, please specify {text box}

10a. How long after birth do you ask for a second sample? (text box)
